# A new mutation c.685G>A:p.E229K in the KCNJ11 gene: A case report of maturity-onset diabetes of the young13

**DOI:** 10.1097/MD.0000000000030668

**Published:** 2022-09-30

**Authors:** Xinjie Song, Yonghong Cao, Jun Ye, Wu Dai, Suwan Zhang, Shuai Ye

**Affiliations:** a Department of Endocrinology, Second People’s Hospital of Hefei City, Hefei City, Anhui Province, China.

**Keywords:** a new mutation, inwardly rectifying subfamily J, maturity-onset diabetes of the young13, member 11

## Abstract

Maturity-onset diabetes of the young (MODY) is an autosomal dominant monogenic diabetes. We report a pair of father and son diagnosed as MODY13 with a new mutation c.685G>A:p.E229K in the inwardly rectifying subfamily J, member 11 (KCNJ11) gene.

**Case presentation:**

A pair of father and son was examined after admission to the hospital and a whole exome test performed. Whole exome test showed that there was a mutation c.685G>A:p.E229K in the KCNJ11 gene encoding a potassium channel, KCNJ11.

**Conclusions:**

The diagnosis of MODY13 requires genetic testing. After confirmation, medication and diet need to be adjusted to control blood glucose. The treatment plan was adjusted. After glimepiride was administered, symptoms of diabetes were effectively improved. According to our knowledge, this is the first reported mutation of c.685G>A:p.E229K in the KCNJ11 gene.

## 1. Introduction

Maturity-onset diabetes of the young (MODY) is an autosomal dominant monogenic diabetes. The disease is characterized by 3 main features: mild hyperglycemia or overt diabetes in at least 3 consecutive generations; onset usually before the age of 25 years; absence of islet autoantibodies and lack of characteristics of type 2 diabetes (i.e., insulin resistance, obesity). The majority of cases with MODY are initially misdiagnosed as type 1 or type 2 diabetes.^[1,2]^ However, thanks to the development of molecular genetics, gene sequencing technology allowed the correct diagnosis and typing of MODY. It is very important to correctly diagnose and type MODY and distinguish it from other types of diabetes since MODY requires precise treatment. At the onset of this study, the Endocrinology Department of Hefei Second People’s Hospital admitted two MODY13 patients – father and son – in whom the gene mutation inwardly rectifying subfamily J, member 11 (KCNJ11), c.685G>A:p.E229K was detected. Here, we report the diagnosis and treatment plan of these 2 patients.

## 2. Case reports

The proband, a 19-year-old male, was admitted to the hospital due to polyuria and polydipsia as well as high blood glucose levels for 5 days. The proband developed polyuria, thirst, and polydipsia without obvious inducements 1 year before admission. The daily water intake was about 3000 mL, and the urine volume was equivalent to the water intake. There was no significant weight loss at that time. Five days before his admission, due to an inflammation of his wisdom teeth, the proband went to the local hospital to check his venous fasting blood glucose, this value was at 18 mmol/L, so he went to our hospital for treatment.

**Figure 1.**
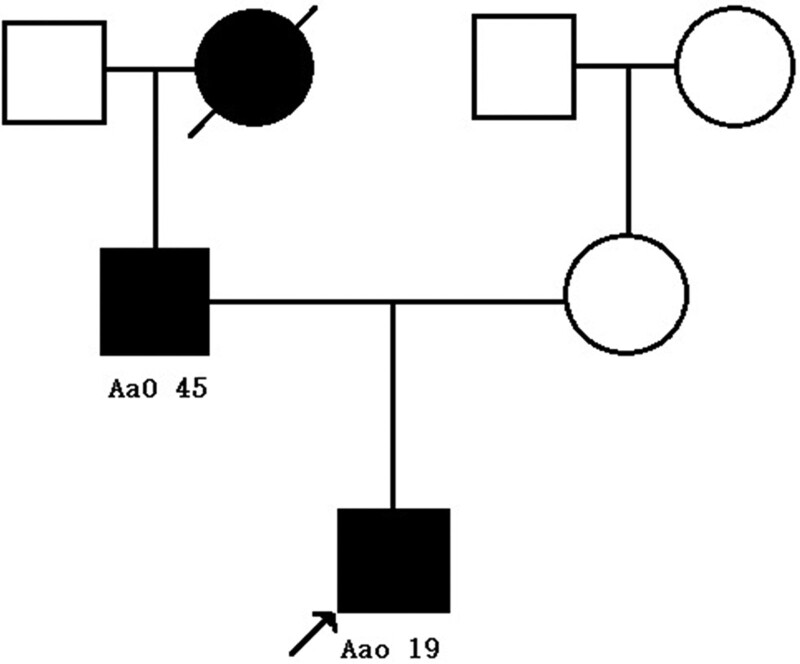
Pedigree showing the prevalence of diabetes and the detected substitutions in the patient’s family. Boxes indicate male subjects; circles indicate female subjects; black boxes/circles indicate the presence of diabetes; oblique line indicates that the respective subject has deceased; arrow points at the patient. AaO = age at onset.

Family history: When admitted to the hospital, the proband said his grandmother was suffering from diabetes and passed away, the condition of her disease was unknown. While the maternal line had no family history of diabetes. A physical examination showed a blood pressure of 132/92 mm Hg, height 175 cm, weight 67 kg, body mass index 21.878 kg/m^2^, well-proportioned body, no black acanthosis in the neck and axilla, no obvious goiter, no abnormalities in the heart, lung or abdomen. The laboratory examination showed urine glucose (3+) and urine ketone body (−). There were no abnormalities in his blood or fecal routines. His liver function, kidney functions, blood lipid, and electrolytes were not abnormal; his thyroid function and hepatitis B check were not abnormal; HbA1c 12.9%, GADA, protein tyrosine phosphatase antibody, IAA, ICA, zinc transporter 8 antibody were negative. The OGTT and synchronous CP release test before and after intensive insulin pump therapy are shown in Table 1 (Remarks: the proband was injected with fast-acting insulin during OGTT before intensive insulin pump therapy). The electrocardiogram was normal; fundus examination showed no abnormalities; CT examination of lungs and liver, gallbladder, pancreas, and spleen showed no abnormalities; ultrasound examination showed double kidney crystals, right kidney cysts, ultrasound examination of carotid artery, lower extremity arteries, ureters, bladder, and prostate showed no abnormalities. According to the electromyogram, the HM latency of the left and right popliteal nerves was prolonged, and the H/M peak-to-peak ratio of the right popliteal nerves reduced. Note, that the family diagram is shown in Figure 1.

**Table 1:** OGTT and synchronous C-Peptide levels of the proband and the proband’s father.

Time	Proband (before therapy)		Proband (after therapy)		Proband’s father	
	PG (mmol/L)	C-P (ng/mL)	PG (mmol/L)	C-P (ng/mL)	PG (mmol/L)	C-P (ng/mL)
0 h	12.43	0.65	8.59	1.33	9.75	1.9
0.5 h	6.38	0.57	/	/	14.86	2.74
1 h	5.17	0.51	/	/	19.74	3.6
2 h	8.58	0.82	11.51	2.64	15.39	4.86
3 h	9.03	0.74	/	/	/	/

The proband’s father, a 45-year-old male, denied polyuria, thirst, polydipsia, and weight loss. He was examined in the outpatient clinic after the proband was hospitalized. The venous fasting blood glucose level was at 12.95 mmol/L, and the blood glucose level 2 hours after a meal was at 19.77 mmol/L, and was subsequently hospitalized treatment. A physical examination showed a blood pressure of 128/82 mm Hg, height 175 cm, weight 82 kg, body mass index 26.78 kg/m^2^, well-proportioned body, no black acanthosis in the neck and axilla, no obvious goiter, no abnormalities in the heart, lung, and abdomen. The laboratory examination resulted in urine glucose (3+) and urine ketone body (−). There were no abnormalities in his blood or fecal routines. His liver function, kidney function, blood lipid, and electrolyte were not abnormal; his thyroid function and hepatitis B check were not abnormal; HbA1c 9.4%, GADA, protein tyrosine phosphatase antibody, IAA, ICA, zinc transporter 8 antibody were negative. The OGTT and synchronous CP release test are shown in Table 1. The electrocardiogram was normal; fundus examination showed no abnormalities; CT examination revealed an old right pulmonary tuberculosis, left lower lobe pulmonary bullae; ultrasound examination showed bilateral carotid atherosclerosis, lower extremity arteriosclerosis, hepatic hemangioma, kidney crystallization, right kidney cyst, prostatic hyperplasia; ultrasound examination of biliary pancreatic spleen, ureter, and bladder were not abnormal. According to the electromyogram the sensory nerve conduction velocity of the right median nerve slowed down slightly, and the HM latency of the tibial nerve of the left and right popliteal fossa was prolonged.

The pancreatic islet function of the proband still existed, and the insulin autoantibodies were all negative. There was no evidence these symptoms were due to an autoimmune damage, and it did not meet the characteristics of type 1 diabetes. The proband was young at onset, was thin, had no insulin resistance, and did not meet the clinical manifestations of typical type 2 diabetes. The presentation of diabetes in 3 consecutive generations in this family prompted us to perform genetic testing. To further clarify whether this abnormal β-cell function was caused by a gene defect, a whole exome test was performed.

**Figure 2.**
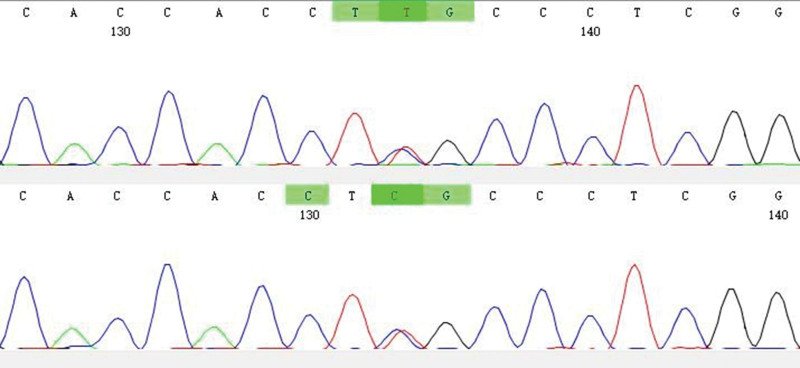
Heterozygous variation in KCNJ11 Gene of the proband. KCNJ11 = inwardly rectifying subfamily J, member 11.

**Figure 3.**
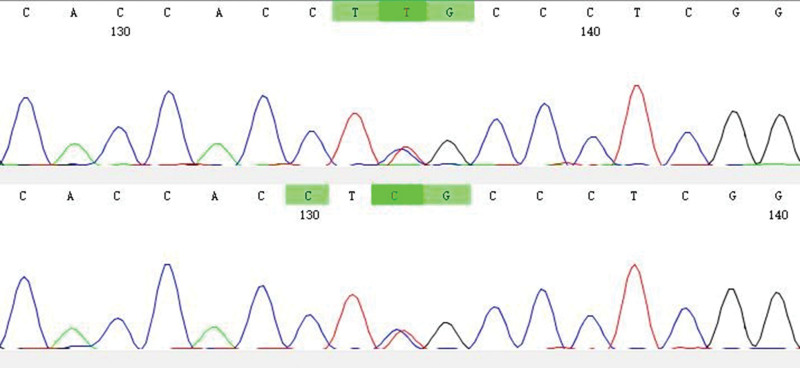
Heterozygous variation in KCNJ11 Gene of the proband’s father. KCNJ11 = inwardly rectifying subfamily J, member 11.

We went through a professional database (Crowd mutation frequency database: 1000 Genomes, ESP, ExAC, gnomAD, etc; locus and disease database: dbSNP, OMIM, HGMD, ClinVar, Decipher, DGV, etc; Health information prediction software: SIFT, Polyphen2, LRT, MutationTaster, FATHMM, M-CAP, CADD, REVEL, dbscSNV, SpliceAI, etc) and biometric prediction software, as well as Wickhams own local database and analysis software. The interpretation rules of sequence variation data refer to the American College of Medical Genetics and Genomics genetic variation classification standards and guidelines.^[3]^ Additionally, the ClinGen sequence variation interpretation expert group has successively released A series of general recommendations and rules (such as PVS1, PS2/PM6, PS3, BA1, PM3, PP5, BP6, etc), as well as an interpretation of specific genes (such as PTEN, CDH1, PAH, etc) and diseases (such as Rasopathy, deafness, etc) guide for further detailed interpretation. The interpretation rules of copy number variation refer to the 2019 version of the American College of Medical Genetics and Genomics Guide for Interpretation and Reporting of Copy Number Variation.^[4]^ Hence, our results for the genetic analysis were as follows: Nuclear genome test detected a heterozygous missense mutation of c.685G>A:p.E229K in the KCNJ11 gene of the subject. However, this mutation was not included in these databases yet. Hence, we claim that this is the first report of the mutation c.685G>A:p.E229K in the KCNJ11 gene. This mutation is the 685th base G of cDNA replaced with A. As a result, the 229th codon encodes glutamic acid instead lysine. Results from second-generation sequencing and first-generation sequencing showed that the mutation was inherited from the father. Notably, this variant is pathogenic (Table 2 and Figs. 2 and 3).^[5–8]^

**Table 2:** Nuclear genome test results.

Gene	Chromosome position	dbSNP ID	Variation naming	Crowd frequency	Variation rating	Zygote type	Relative monitoring results	
							Father	Mother
KCNJ11	chr11:17408954	rs587783673	KCNJ11:NM_000525:exonl:c.685G>A:p.E229K	Not included	Pathogenic	Hybrid	Hybrid	/

KCNJ11 = inwardly rectifying subfamily J, member 11.

Analyzing the mitochondrial genome showed that no pathogenic/probable pathogenic variants, that are highly related to the clinical phenotype of the subject, were found on the mitochondrial genome (Table 3).

**Table 3:** No pathogenic/probable pathogenic variants that are highly correlated with the clinical phenotype of the subject were found on the mitochondrial genome.

Gene	Genome location	dbSNP ID	Variation naming	Variation type	Variation abundance	Variation classification	Relative monitoring results	
							Father	Mother
/	/	/	/	/	/	/	/	/

**Figure 4.**
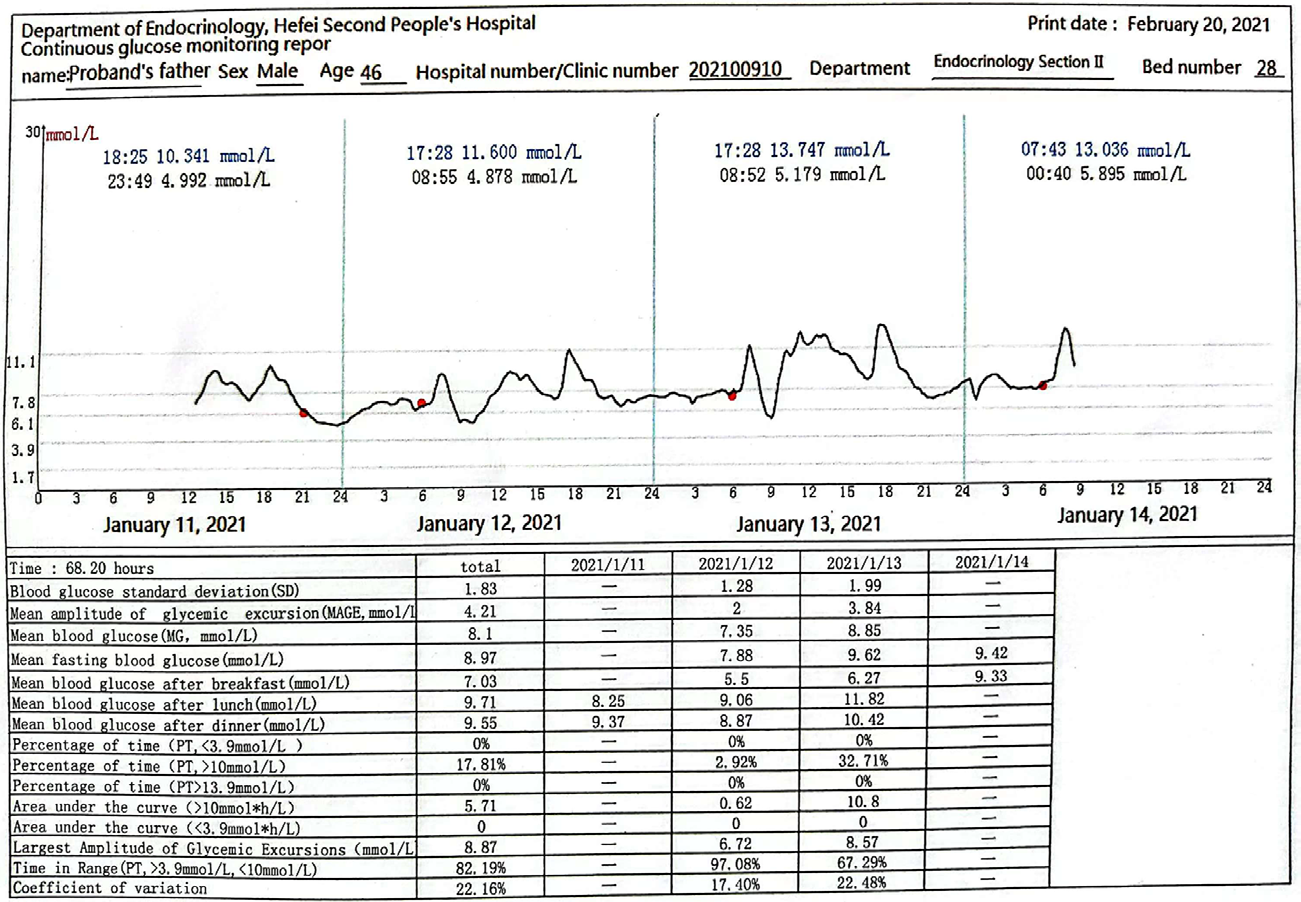
Real-time dynamic blood glucose monitoring report of the proband’s father.

**Figure 5.**
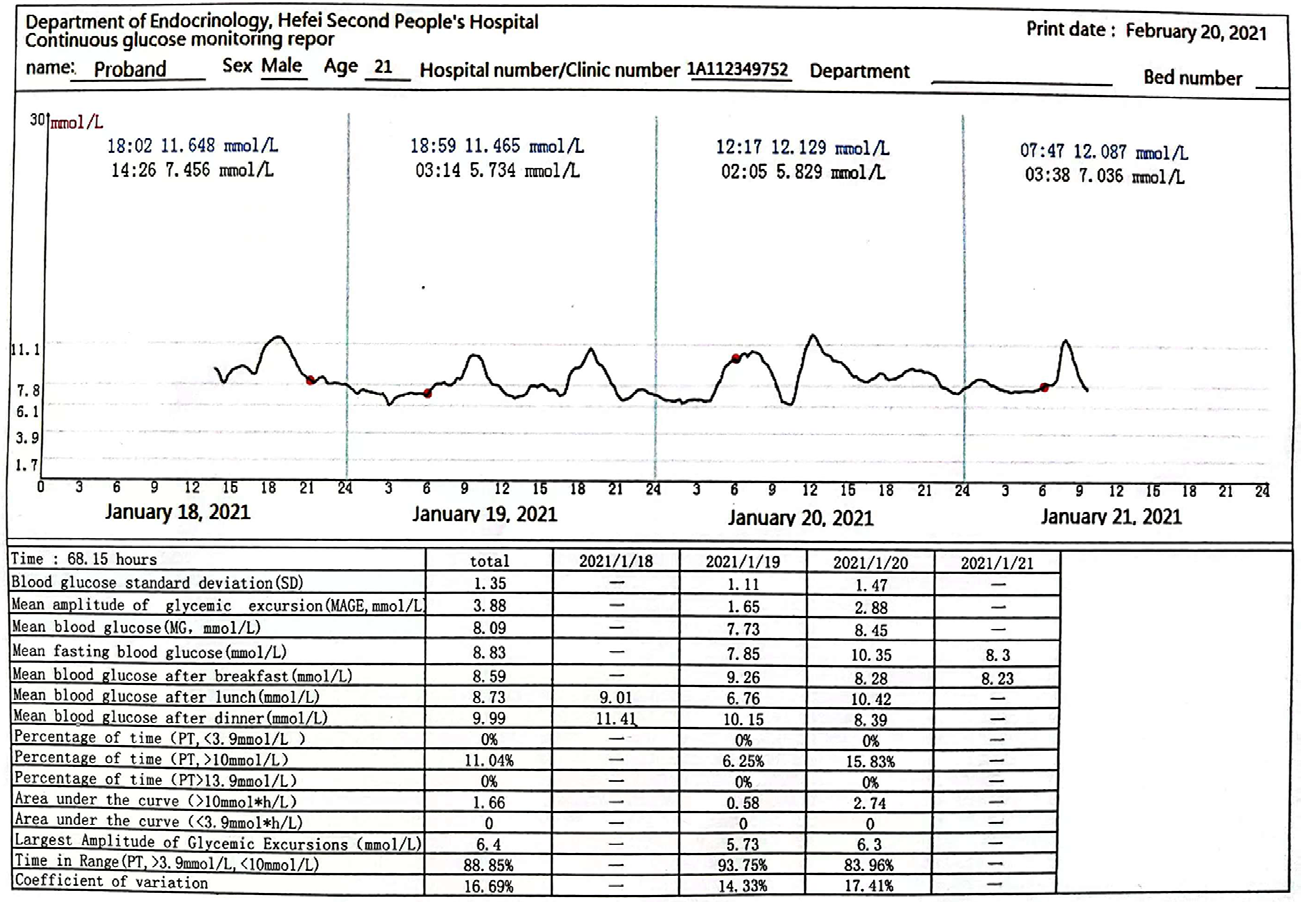
Real-time dynamic blood glucose monitoring report of the proband.

On the basis of lifestyle changes, the proband was given intensive insulin pump therapy (the maximum dose of insulin throughout the day is 50.6 U/d). Hence the blood glucose level was reduced compared to before but did not reach the target. On the 11th day of insulin pump treatment, it was substituted with 4 subcutaneous injections of insulin glargine combined with insulin glulisine to continue to strengthen hypoglycemic, and was discharged from the hospital with this plan, the fasting blood glucose at discharge was 6.2 to 6.7 mmol/L, and the blood glucose 2 hours after the meal was 7.8 to 13.1 mmol/L. The father of the proband was subsequently hospitalized. During his hospitalization, he was directly given glimepiride 2 mg/d orally. The blood glucose was monitored gradually with the real-time dynamic blood glucose monitoring system following for 3 days. The TIR was at 82.19% (3.9–10 mmol/L) (Fig. 4), and the proband was discharged from the hospital with glimepiride levels of 2 mg/d. The fasting blood glucose was 6 to 7 mmo/L at discharge, and the blood glucose 2 hours after a meal was 6 to 9 mmol/L. After the proband’s father got a good effect, on the 59th day of his insulin use, glimepiride 1 mg/d was administered orally in the outpatient clinic. While the insulin dosage was gradually reduced, the blood glucose was monitored gradually with the real-time dynamic blood glucose monitoring system following for 3 days. The TIR was 82.19% (3.9–10 mmol/L) (Fig. 5). One week later, glimepiride was added to 2 mg/d, insulin stopped. The fasting blood glucose at that point was 5.1 to 6.4 mmol/L, and the blood glucose 2 hours after a meal 5.3 to 8.6 mmol/L.

All procedures performed in studies involving human participants were in accordance with the ethical standards of the institutional and/or national research committee and with the 1964 Helsinki declaration and its later amendments or comparable ethical standards.

## 3. Discussion

MODY is an autosomal dominant monogenic diabetes. Currently, the commonly used diagnostic criteria for MODY are: at least 1 to 2 patients in the family have a disease onset before the age of 25; at least 3 consecutive generations of autosomal dominant inheritance; generally no insulin treatment required within 5 years after diagnosis; β-cell dysfunction.^[9,10]^ The clinical manifestations of MODY are complex while there is also clinical heterogeneity. In recent years, it has been gradually discovered that several MODY patients do not fully meet the above criteria. To unambiguously diagnose patients, genetic testing is needed in addition to deep understanding of the various types of MODY. At present, there are 14 types of MODY genes reported in the online human Mendelian genetic database, namely MODY1/HNF4α,MODY2/GCK,MODY3/HNF1α,MODY4/PDX1,MODY5/HNF-1β,MODY6/NEUROD1,MODY7/KLF11,MODY8/CEL,MODY9/PAX4,MODY10/INS,MODY11/BLK,MODY12/ABCC8,MODY13/KCNJ11,MODY14/APPL1.^[11–13]^ All currently known subtypes of MODY are caused by dominantly acting heterozygous mutations in genes important for the development or function of β-cells.

One study in 2012 based on WES, high-throughput multiplex genotyping and linkage analysis, unambiguously identified a 13th MODY gene. This gene KCNJ11 encodes a pore-forming KIR6.2 subunit of the ATP-dependent potassium (K-ATP) channel in pancreatic β-cells.^[14]^ The K-ATP channel is a hetero-octamer consisting of 4 KIR6.2 subunits and 4 sulfonylurea receptor 1 subunits (encoded by ABCC8). Subunits and 4 sulfonylurea receptor 1 links cellular nutrient metabolism to membrane electrical activity by regulating K + fluxes across the membrane. In β-cells, the K-ATP channel contributes to glucose homeostasis by regulating insulin in response to fluctuations of the plasma glucose level. These mutations in the KCNJ11 gene shift the probability of the KATP channels to be active and contain an open configuration causing a plasma membrane hyperpolarization and thereby inhibiting insulin secretion.^[15–17]^ These gene mutations in KCNJ11 can cause neonatal diabetes mellitus, impaired fasting glucose, impaired glucose tolerance, congenital hyperinsulinism, or MODY13.^[18–20]^

In patients with type 2 diabetes, sulfonylurea drugs are associated with hypoglycemia and failure to durably maintain improvements in glucose control. Also, these drugs have been associated with an increased all-cause mortality and cardiovascular events in adults with type 2 diabetes.^[21,22]^ Therefore, the clinical use rate of sulfonylureas is low, yet sulfonylurea drugs are the preferred choice to treat MODY13. Notably, sulfonylurea drugs can restore the blockade of the channel function that is caused by this mutation. This simple and low-cost treatment allows most patients to achieve an improved glycemic control – with minimal hypoglycemia – compared to insulin injections.^[23]^ Pamela Bowman and colleagues studied a long-term follow-up of an international cohort of patients with neonatal diabetes due to KCNJ11 mutations who switched their treatments from insulin to sulfonylurea. After about 10 years of follow-up, 75 (93%) of 81 participants remained on the sulfonylurea therapy alone. Very good glycemic control was maintained for patients for whom paired data were available on HbA1c and sulfonylurea at all time points (i.e., pre-transfer [for HbA1c], year 1, and most recent follow-up; n = 64), with median HbA1c 6.4% at most recent follow-up, compared to 5.9% at 1 year and 8.1% before transfer to sulfonylurea therapy. In the whole cohort of 809 patients, there were no reports of severe hypoglycemia even years of follow-up. It could be that there is continuous improvement of the capacity of β-cells for glucose-stimulated insulin secretion during sulfonylurea treatment.^[24]^

In conclusion, molecular genetic diagnosis can provide patients with a more reasonable treatment plan, improve the quality of life and clinical outcome, and enable their family members to receive early diagnosis and reasonable treatment. In this case, the proband had an early-onset diabetes, no history of neonatal hypoglycemia, no history of ketosis, negative insulin-related antibodies, and no obvious insulin resistance. The initial blood glucose control of insulin was not satisfactory, and the blood glucose level gradually reached the standard after administering sulfonylureas. At the current follow-up, sulfonylureas are used alone, and the blood sugar level is well controlled. At the same time, the father of the proband only took sulfonylureas, and his blood sugar level was well controlled. The above characteristics are consistent with the MODY13 type reported in the literature. To our knowledge this is the first reported mutation c.685G>A:p.E229K in the KCNJ11 gene in Asian population. We further found that for MODY patients, early intensive insulin therapy is also effective in improving islet function. The here described case suggests that children or young-onset diabetes patients with a family genetic background should consider screening for MODY. Due to the early age of onset and insufficient insulin secretion, these patients are easily misdiagnosed as type 1 diabetes or type 2 diabetes. It is necessary to pay attention to the traceability of their family histories and clinical characteristics of family members. Hence, relevant genetic testing should be performed when necessary. In the future, more relevant follow-up studies are needed, and should provide more guidance and suggestions for early diagnosis and reasonable treatment of MODY.

## Author contributions

Conceptualization: Xinjie Song.

Data curation: Yonghong Cao, Jun Ye, Shuai Ye.

Formal analysis: Xinjie Song.

Investigation: Yonghong Cao, Wu Dai.

Methodology: Xinjie Song.

Resources: Jun Ye.

Software: Wu Dai, Suwan Zhang, Shuai Ye.

Validation: Suwan Zhang.

Writing – original draft: Xinjie Song.

Writing – review & editing: Xinjie Song, Yonghong Cao.
